# Childhood *Mycoplasma pneumoniae*: epidemiology and manifestation in Northeast and Inner Mongolia, China

**DOI:** 10.1128/spectrum.00097-24

**Published:** 2024-04-12

**Authors:** Fei Wang, Qi Cheng, Hongying Duo, Jichun Wang, Jingjing Yang, Shujun Jing, Jing Li, Xiandong Zhou, Yunxiao Shang, Ning Chen, Zhiliang Tian, Han Zhang, Xuxu Cai

**Affiliations:** 1 Department of Pediatrics, Shengjing Hospital of China Medical University, Shenyang, Liaoning, China; 2 Department of Pediatrics, The Affiliated Hospital of Inner Mongolia Medical University, Hohhot, Inner Mongolia, China; 3 Diagnosis and Treatment Center for Children, The Affiliated Hospital of Changchun University of Chinese Medicine, Changchun, Jilin, China; 4 Department of Respiratory Medicine, Dalian Women and Children’s Medical Center Group, Dalian, Liaoning, China; 5 Department of Pediatrics, The First Affiliated Hospital of Jinzhou Medical University, Jinzhou, Liaoning, China; 6 Department of Pediatrics, The 2nd Affiliated Hospital of Harbin Medical University, Harbin, Heilongjiang, China; Children's National Hospital, George Washington University, Washington, District of Columbia, USA

**Keywords:** *Mycoplasma pneumoniae*, SMPP, bronchoscopic manifestations, age distribution, disease spectrum

## Abstract

*Mycoplasma pneumoniae* (MP) is commonly detected in children. However, the epidemiological trends of MP in Northeast (NE) China are unclear. This retrospective study aimed to investigate the prevalence of MP infections in this understudied region. The clinical manifestations and bronchoscopic findings observed in hospitalized patients with severe *Mycoplasma pneumoniae* pneumonia (SMPP) were collected from comprehensive data obtained from six tertiary hospitals in NE and Inner Mongolian (IM) China, from 1 January 2017 to 31 December 2023. A total of 5,593,530 children who visited the outpatient and emergency departments, and 412,480 inpatient hospitalized children were included in the study. The positivity rate of MP immunoglobulin M (IgM) in the children who visited the outpatient and emergency departments varied from 7.80% to 10.12%, whereas that of MP infection in hospitalized children ranged from 27.18% to 30.10%. Children hospitalized for MP infection were mainly concentrated in the 1- to 4-year (41.39%) and 4- to 7-year (24.25%) age groups. Before 2020, the season with the highest incidence of MP was winter. After the implementation of non-pharmaceutical interventions (NPIs), the MP epidemic season changed, and the number of children with MP infections decreased; however, the proportion of MP infections in hospitalized children did not change significantly. Starting from August 2023, the MP infection rate in outpatient, emergency, and hospitalized children increased sharply, with SMPP and its complications (e.g., plastic bronchitis and pleural effusion) increasing significantly. MP is prevalent in NE and IM, China. When the NPIs ended, MP infection showed a delayed outbreak trend, and the number of children with severe infection increased significantly.

**IMPORTANCE:**

In Northeastern (NE) and Inner Mongolia (IM), the incidence of *Mycoplasma pneumoniae* (MP) infections, including severe *Mycoplasma pneumoniae* pneumonia (SMPP), is high, posing health risks and imposing substantial economic burdens on the local population. Therefore, it is imperative to prioritize the study of MP prevalence and address the research gaps in MP epidemiology in these areas of China. We obtained a comprehensive collection of pediatric outpatient, emergency, and inpatient data from six public Grade III hospitals. We believe that our study makes a significant contribution to the literature because understanding regional variations in MP infections can help healthcare professionals tailor prevention and treatment strategies, and studying bronchoscopic manifestations can provide insights into the impact of the disease on the respiratory system, potentially leading to a more effective clinical management.

## INTRODUCTION

*Mycoplasma pneumoniae* (MP), the smallest prokaryotic cell-type microorganism, is characterized by a cell structure devoid of cell walls, rendering it an intermediary between bacteria and viruses. It is a significant etiological factor in respiratory tract infections in children, ranging from mild to life-threatening (1). MP infections can occur year-round, following an epidemic cycle of approximately 3–7 years, with each epidemic spanning 1–2 years (2). MP was the most commonly detected microorganism among children aged ≥5 years who were hospitalized with community-acquired pneumonia in an epidemiological study in the USA (2). *Mycoplasma pneumoniae* pneumonia (MPP) accounts for 30%–50% of childhood pneumonia cases in epidemic peak years according to a study in Henan, China (3). The seasonal characteristics of MP infections are closely related to the living environment, public health measures, and activity patterns. A global survey of MP detection at separate sites in 21 countries between April 2017 and March 2021 indicated a general decline in MP cases relative to the preceding 3 years (4). This decline coincided with the implementation of non-pharmaceutical interventions (NPIs) against coronavirus disease 2019 (COVID-19) in March 2020. The data showed that the reopening of schools had a minimal effect on MP transmission in 2020 (4), which is at odds with the prevailing conjecture that children primarily drive MP infection (5).

MP is widely prevalent in China, with distinctions in the northwestern, southern, central, and northeastern areas of the country. In the Northeast (NE) and Inner Mongolian (IM) areas, the incidence of MP infections, including severe *Mycoplasma pneumoniae* pneumonia (SMPP), is notably high, posing health risks and increasing the economic burden on the population. The NE and IM regions are consistent in dimensions, with the area covering 37°N and 53°N in China. However, research on the epidemiological trends of MP is still lacking. Moreover, the impact on the epidemiology of MP before and after the epidemic has not been reported for NE China. Therefore, prioritizing the study of MP prevalence and addressing the research gaps in MP epidemiology in the NE and IM regions of China is crucial.

This study analyzed pediatric outpatient, emergency, and inpatient data from six public Grade III hospitals in four provinces of NE and IM China, between 2017 and 2023. Data were collected within the context of investigating MP prevalence in the three NE provinces and IM of China. The respiratory disease spectrum and complications of SMPP in hospitalized patients with MP infections were further analyzed. Some patients with SMPP required bronchoscopy as part of their treatment; therefore, changes observed in patients with SMPP who underwent bronchoscopy were also assessed.

## RESULTS

**Fig 1:**
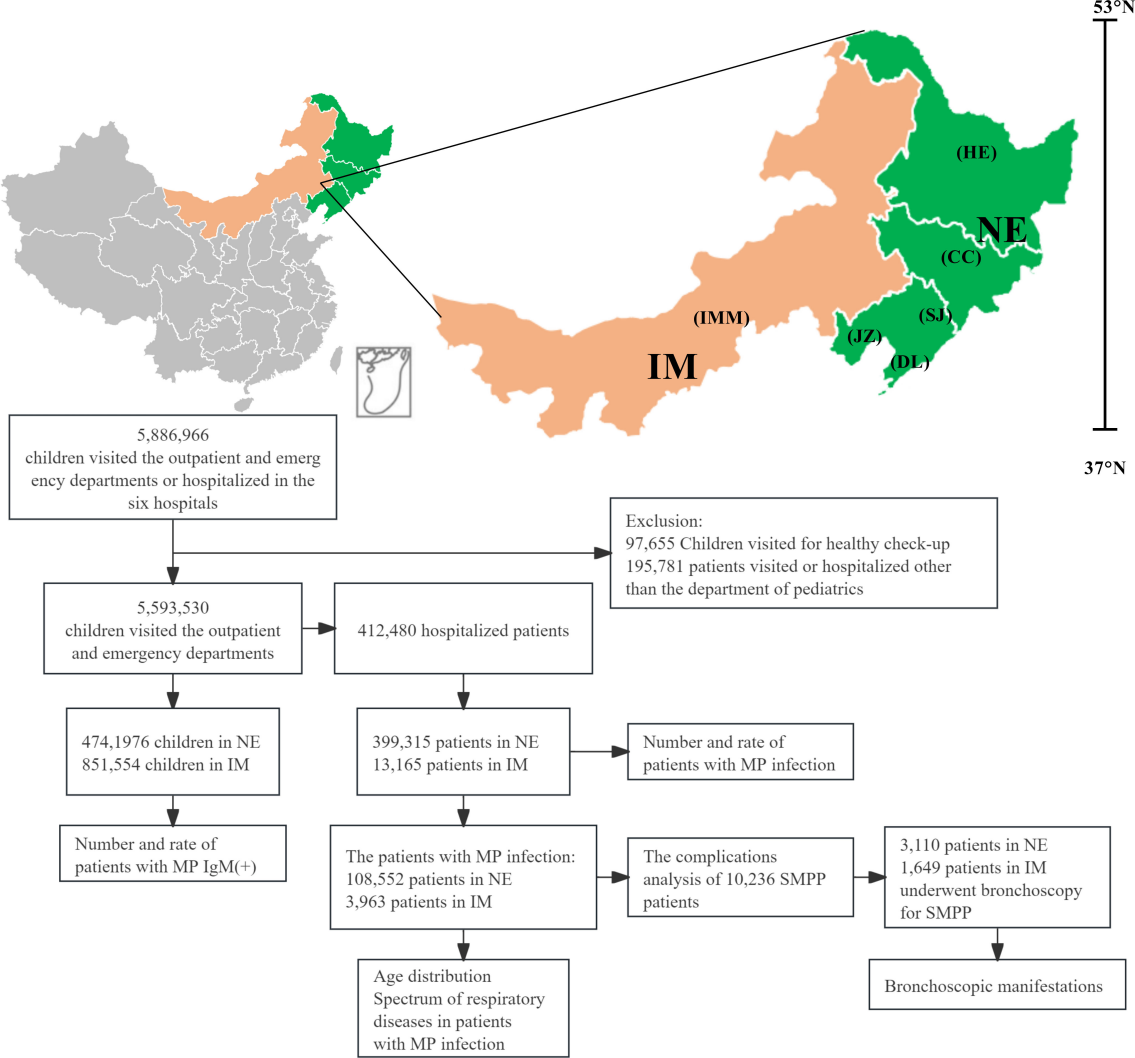
Study flow chart. Of the 5,886,966 patients from NE and IM who visited the hospitals between 1 January 2017 and 31 December 2023, 5,593,530 were included in this study, of which, 412,480 were hospitalized. The number and rate of outpatients with MP IgM(+) were also studied. Age distribution, spectrum of respiratory diseases, complications, and bronchoscopic manifestations of SMPP in inpatients were analyzed. The map is created by the authors and based on the "Territory of the People's Republic of China" at the website of the Ministry of Natural Resources (https://www.gov.cn/guoqing/2017-07/28/content_5043915.htm). NE, Northeast region of China; IM, Inner Mongolian region in China; SJ, Shengjing Hospital of China Medical University (National Center for Children’s Health, Northeast Region); DL, Dalian Women and Children’s Medical Center Group; JZ, The First Affiliated Hospital of Jinzhou Medical University; CC, The Affiliated Hospital of Changchun University of Chinese Medicine; HE: The 2nd Affiliated Hospital of Harbin Medical University; IMM, The Affiliated Hospital of Inner Mongolia Medical University.

From 1 January 2017 to 31 December 2023, the pediatric departments of six public tertiary hospitals in the NE and IM areas in China received 5,886,966 patients. For this study, 97,655 and 195,781 patients were excluded for undergoing routine health examinations and for being treated or hospitalized outside of pediatrics, respectively. The remaining 5,593,530 children from the outpatient and emergency departments were included in this study; of these children, 412,480 were admitted for treatment (Fig. 1).

### Positivity rate of MP IgM in children who visited the outpatient and emergency departments

**Fig 2:**
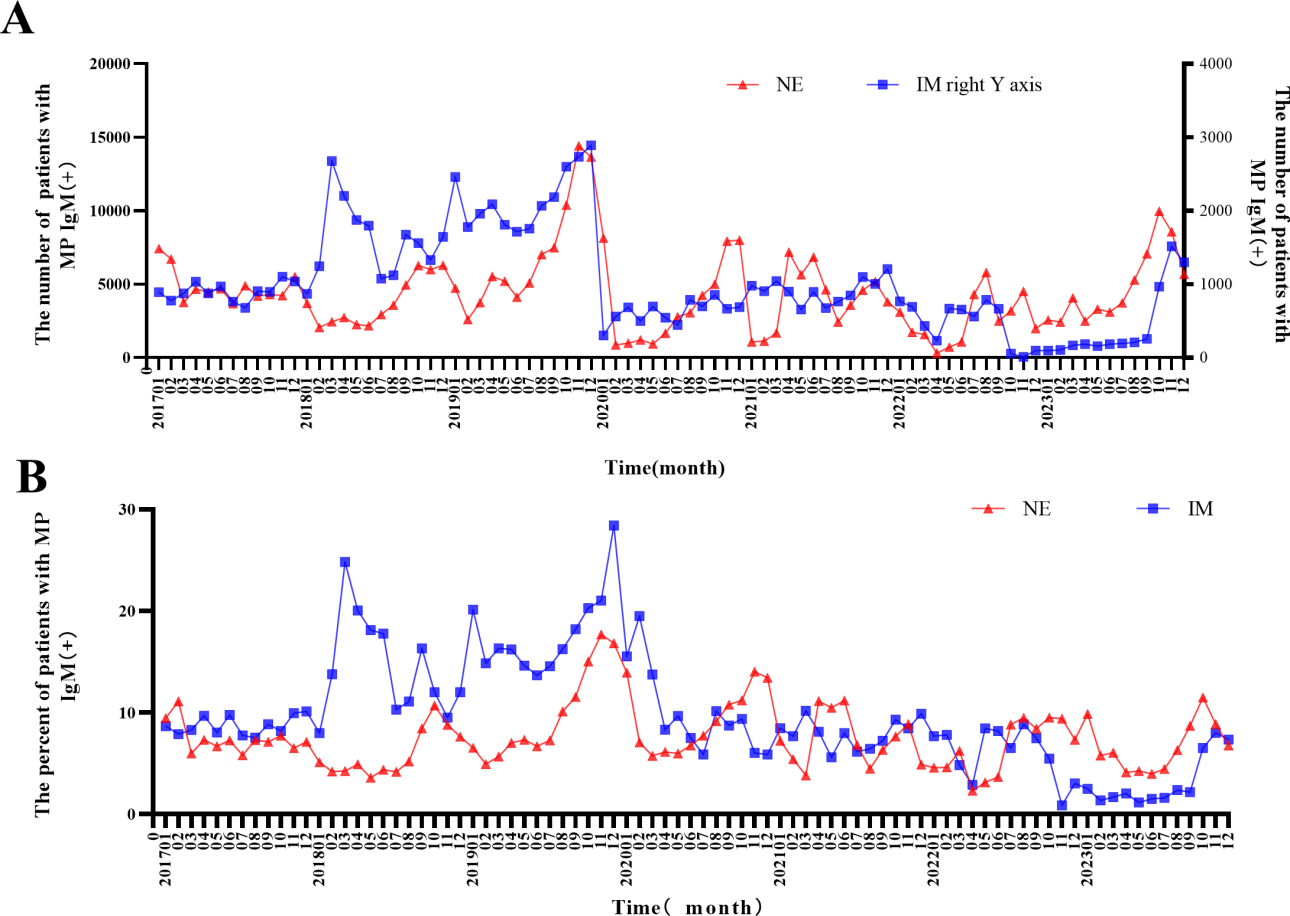
Number and proportion of MP IgM(+) children who visited the outpatient and emergency departments in different hospitals. (**A**) Number of MP IgM(+) children who visited the outpatient and emergency departments. (**B**) Positivity rate of MP IgM(+) in children who visited the outpatient and emergency departments.

The 5,593,530 children included in the study were distributed as follows: 4,741,976 in NE and 851,554 in IM. The number and percentage of MP immunoglobulin M (IgM)(+) cases were counted per month to assess trends in MP prevalence (Fig. 2). The number of MP IgM(+) cases among children who visited the outpatient and emergency departments was 369,806 in NE and 85,300 in IM, with mean positivity rates of 7.80% for NE and 10.12% for IM. The number and positivity rate of MP IgM(+) cases increased significantly in winter, decreased in summer and autumn, and peaked in 2019 and 2023. The number of MP IgM(+) cases decreased from January 2020 to December 2022, and following the cessation of NPIs, the number and positivity rate of MP IgM(+) cases in the outpatient and emergency departments remained low until August 2023. During 2017‒2019 and 2020‒2022, the mean number (percentage) of MP IgM(+) cases each year was 81,318 (8.77%) and 49,459 (8.23%), respectively. By 2023, it was 63,634 (6.32%) (Table 1); in October 2023, it was 10.78%, indicating an outbreak.

**TABLE 1:** Information of the patients analyzed in the 2017‒2019, 2020‒2022, and 2023 groups^*a*^

	2017–2019	2020–2022	2023		
	*n* (%)	*n* (%)	*n* (%)	χ2	*P*
Patients with MP IgM(+) in the outpatient and emergency departments per year	81,318 (8.77)	49,459 (8.23)	63,634 (6.32)		
Patients with MP infection in the inpatient departments per year	19,501 (28.07)	11,933 (25.52)	10,273 (28.55)		
Age distribution of hospitalized children with MP infection					
0-to 1-year	1,609 (8.25)_a_	1,143 (9.56)_b_	1,482 (8.14)_a_	235.917	<0.001
1- to 4-year	7,814 (40.07)_a_	4,985 (41.72)_b_	8,173 (44.87)_c_		
4- to 7-year	5,072 (26.01)_a_	2,547 (21.31)_b_	4,433 (24.34)_c_		
7- to 10-year	3,252 (16.68)_a_	1,958 (16.38)_a_	2,607 (14.31)_b_		
10- to 14-year	1,754 (8.99)_a_	1,317 (11.02)_b_	1,518 (8.33)_a_		
Disease spectrum distribution of hospitalized children with MP infection					
Bronchopneumonia	5,628 (58.88)_a_	3,285 (66.06)_b_	5,910 (49.53)_c_	1,476.731	0.001
Lobar pneumonia	1,585 (16.58)_a_	423 (8.51)_b_	2,565 (21.50)_c_		
SMPP	1,878 (19.65)_a_	577 (11.60)_b_	2,869 (24.04)_c_		
Asthma	226 (2.36)_a_	468 (9.41)_b_	252 (2.11)_a_		
Bronchiolitis	173 (1.81)_a_	187 (3.76)_b_	213 (1.79)_a_		
Pertussis-like syndrome	69 (0.72)_a_	33 (0.66)_a_	123 (1.03)_a_		

^*a*^ The average number and percentage of each age group per year were calculated; cross-tabulation statistical analysis was performed; and statistically significant differences were observed. The Bonferroni method was used for pairwise comparisons, and the differences between any two groups are shown. Each subscript (a, b, c) indicates a subset of the year category; the column proportions of these categories do not differ significantly from each other at the 0.05 level.

The seasons with the highest incidence changed slightly during the implementation of NPIs. Before January 2020, the seasons with the highest MP incidence were winter and spring (November–February and March of the following year). However, after January 2020, the seasons with the highest MP incidence were summer and autumn (August–December 2020, March–July 2021, and July–October 2022).

### MP infection in hospitalized children

#### Proportion of MP infection in hospitalized children

**Fig 3:**
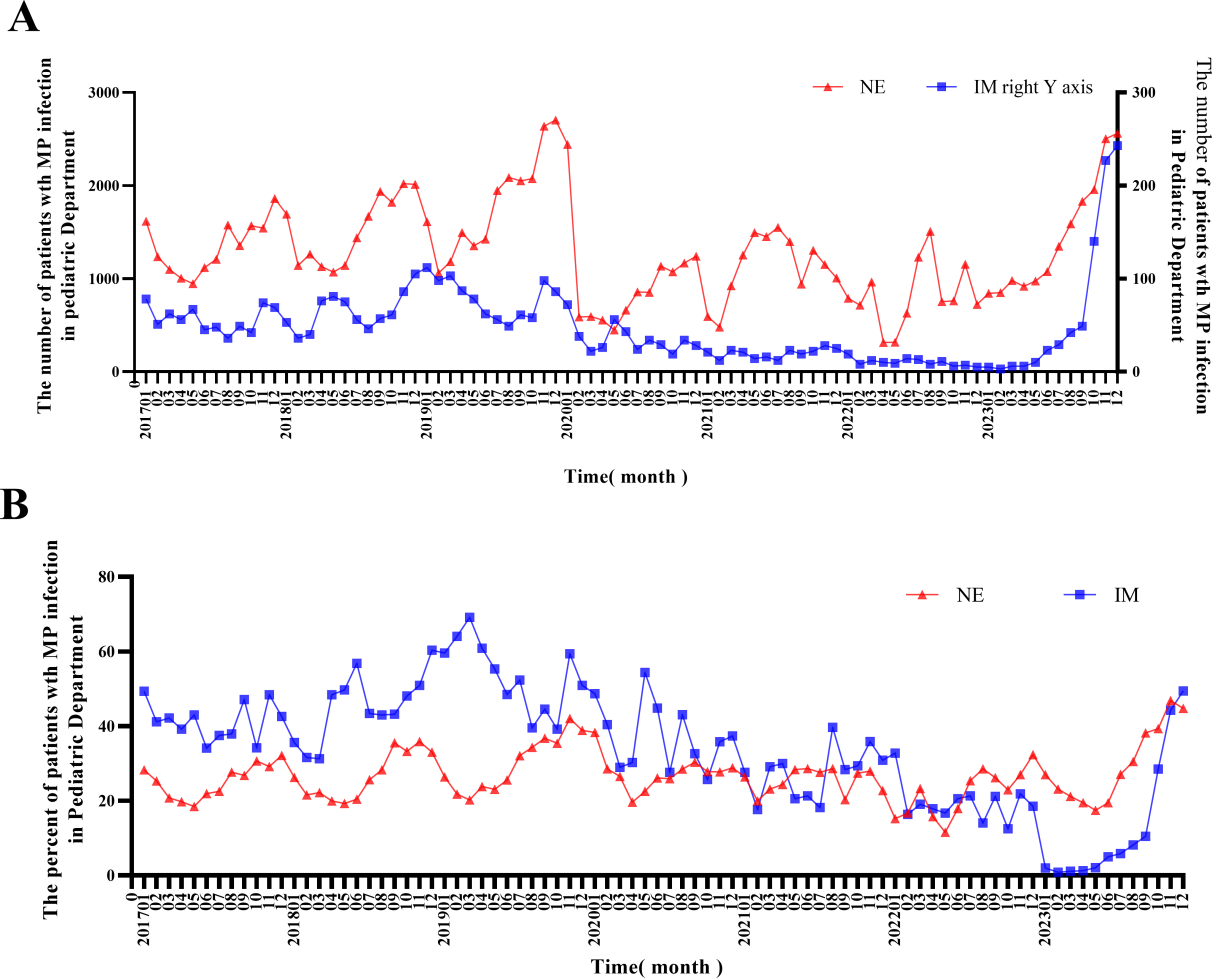
Number and proportion of MP infections in hospitalized children. (**A**) Number of MP infections in hospitalized children. (**B**) Proportion of MP infections in hospitalized children.

The number of children hospitalized between 1 January 2017 and 31 December 2023 was 412,480, with 399,315 children hospitalized in NE and 13,165 hospitalized in IM. The total number of hospitalized and MP-infected children per month was counted to assess the prevalence of MP infection among hospitalized children (Fig. 3). A total of 112,515 children had MP infections (108,552 in NE and 3,963 in IM). The mean positive rates of MP infection in hospitalized children were 27.18% and 30.10% for NE and IM, respectively. The number of hospitalized children infected with MP was the highest in 2019 and 2023. After January 2020, the number of hospitalized children with MP infection decreased significantly, persisting until August and September 2023. The seasons with the highest incidence changed significantly during the implementation of NPIs. During 2017‒2019 and 2020‒2022, the peak seasons of MP infection among hospitalized children were autumn and winter (October to February of the second year) and summer and autumn (June to October), respectively. Analysis of the proportion of MP infections in hospitalized children revealed no significant changes before and after January 2020. The mean number (percentage) during the year was 19,501 (28.07%) from January 2017 to December 2019, 11,933 (25.52%) from January 2020 to December 2022, and 18,213 (28.55%) in 2023 (Table 1). Notably, since July 2023, the number of children hospitalized for MP infections increased significantly. Since September 2023, the number of hospitalized children with MP infection has increased significantly, with a total of 1,878 children, accounting for 35.72% in September.

#### Age distribution of hospitalized children with MP infection

**Fig 4:**
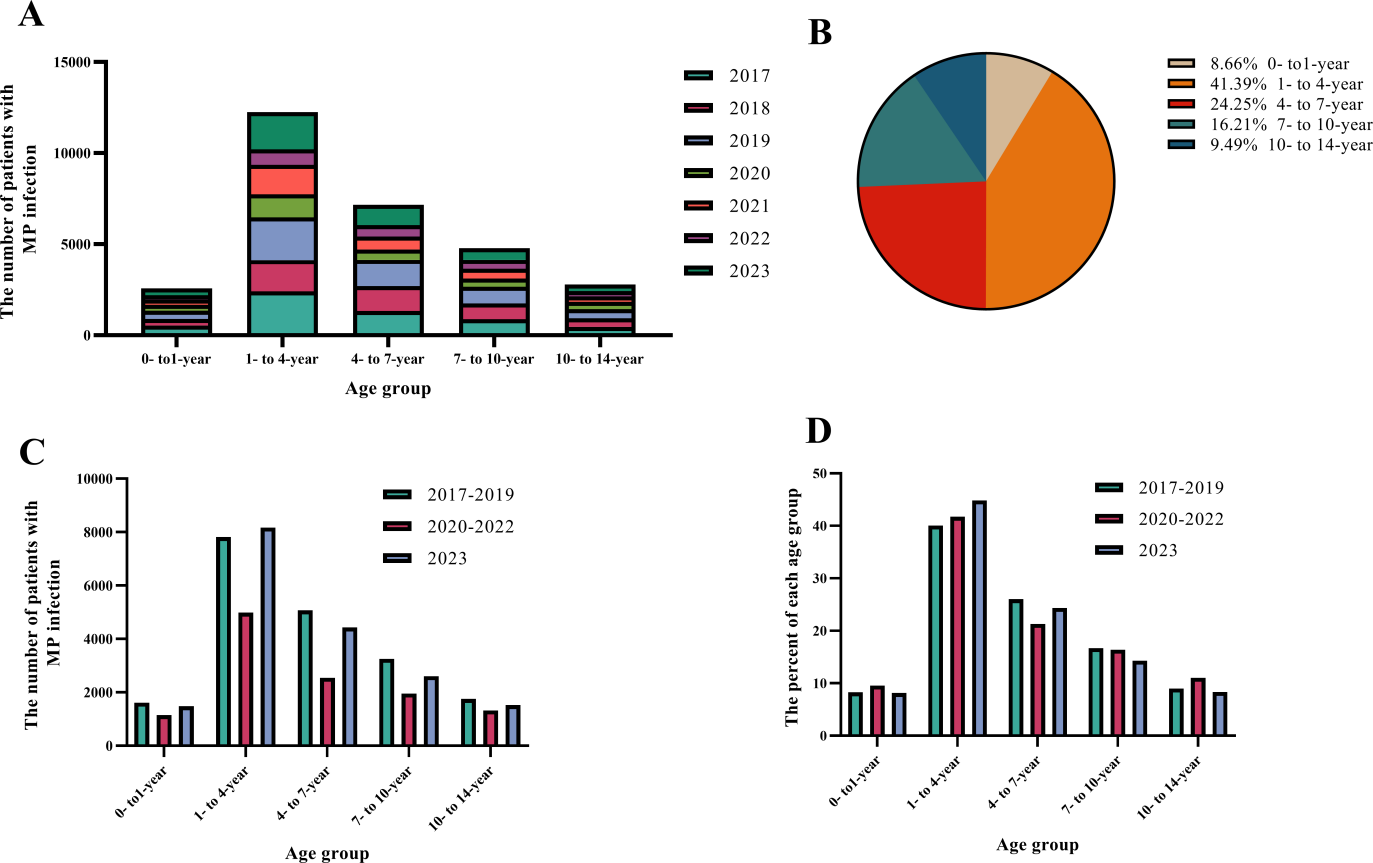
Age distribution of hospitalized children with MP infection. (**A**) Number of hospitalized children of different age groups in NE and IM. (**B**) Proportion of hospitalized children of different age groups. (**C**) Number of hospitalized children of different age groups in the 2017‒2019, 2020‒2022, and 2023 groups. (**D**) Proportion of hospitalized children of different age groups in the 2017‒2019, 2020‒2022, and 2023 groups.

A total of 112,515 patients were hospitalized with MP infection during the study period. The inpatients were divided into five age groups, and the numbers and proportions of each age group were calculated by year: 0- to 1-year (9,739, 8.66%), 1- to 4-year (46,572, 41.39%), 4- to 7-year (27,289, 24.25%), 7- to 10-year (18,237, 16.21%), and 10- to 14-year (10,678, 9.49%) (Fig. 4). The 1- to 4-year and 4- to 7-year age groups accounted for most hospitalizations due to MP infection. The hospitalized patients were further divided according to hospitalization time: 2017–2019, 2020–2022, and 2023. The average number and percentage of each age group per year were calculated, and cross-tabulation statistical analysis was performed. Statistically significant differences were observed in the overall composition of each region among the five age groups (Table 1). The Bonferroni method was used for pairwise comparisons, and differences between any two age groups were considered statistically significant at *P* < 0.05 (Table 1). The age distribution and proportion of children were analyzed based on age and hospitalization time (Fig. 4). During the 2020–2022 period, among the hospitalized children with MP infection, the number of children in the 0- to 1- and 10- to 14-year-old groups increased. During 2023, the number and percentage of the 1- to 4-year-old group increased to 8,173 and 44.87%, respectively, accounting for most hospitalizations.

#### Disease spectrum of MP infection in hospitalized children

**Fig 5:**
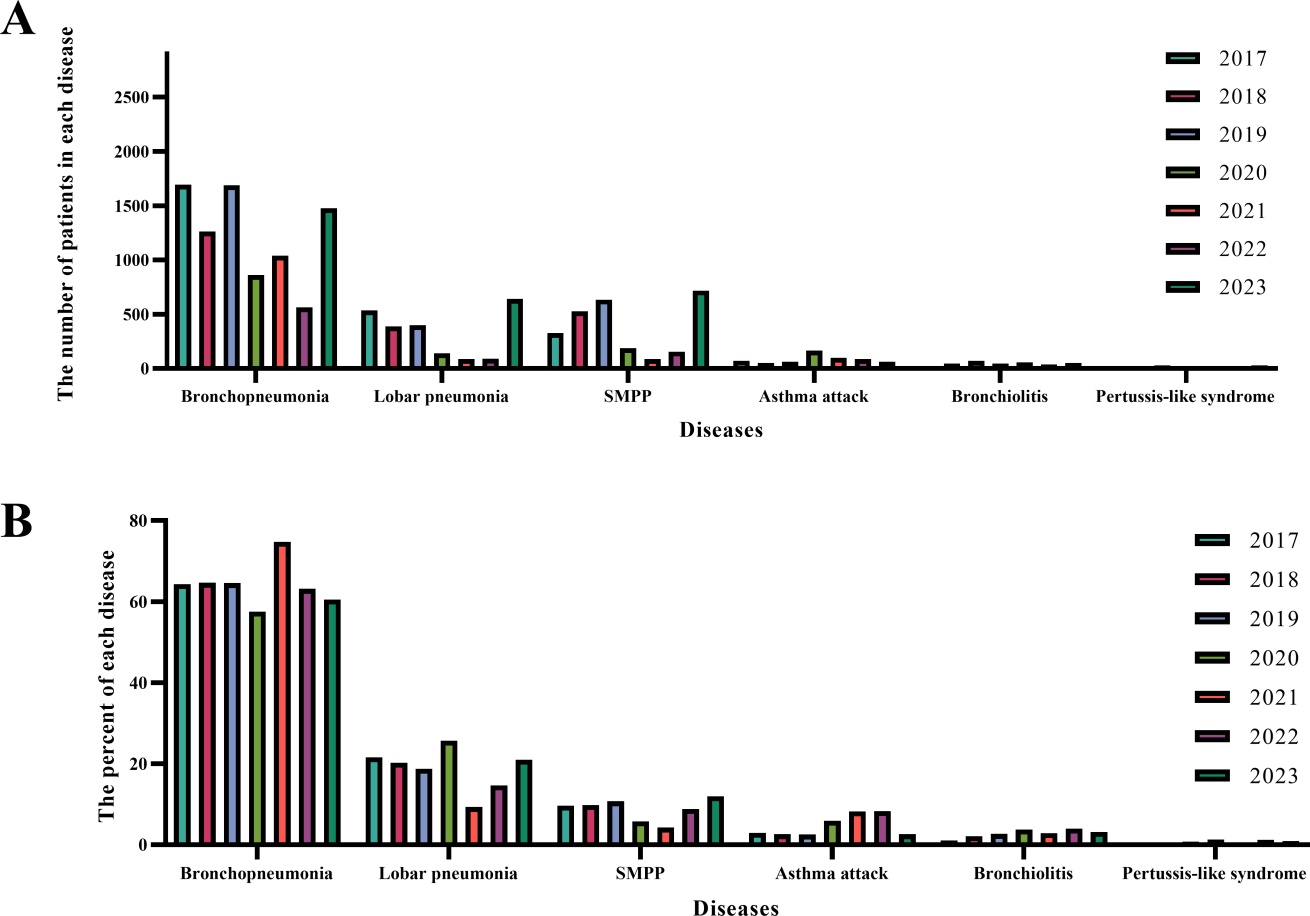
Disease spectrum of MP infection in hospitalized children. (**A**) Number of children with each disease spectrum. (**B**) Proportion of each disease spectrum.

Because MP is mainly transmitted through respiratory droplets, its major effects are observed in the respiratory tract. Therefore, this study analyzed alterations in the prevalence of various respiratory diseases among hospitalized patients with MP infection. These diseases include bronchopneumonia, lobar pneumonia, SMPP, asthma attacks, bronchiolitis, and pertussis-like syndrome (Fig. 5). The total number of patients diagnosed with respiratory diseases was 55,527, with the total number and percentage of each disease being 32,647 (58.79%) for bronchopneumonia, 8,590 (15.47%) for lobar pneumonia, 10,236 (18.43%) for SMPP, 2,334 (4.20%) for asthma attacks, 1,293 (2.33%) for bronchiolitis, and 427 (0.77%) for pertussis-like syndrome (Table 1). Bronchopneumonia was the main manifestation of MP infection. The number of patients with bronchopneumonia, lobar pneumonia, SMPP, bronchiolitis, and pertussis-like syndrome declined sharply from 2020 to 2022, and increased in 2023.

The average number and percentage of each disease per year were then calculated according to the hospitalization time: 2017–2019, 2020–2022, and 2023. Cross-tabulation statistical analysis was performed, and statistically significant differences were observed in the composition of each disease (Table 1). The percentage of each disease was affected by the implementation of NPIs during the 2020–2022 period: the percentages of lobar pneumonia (8.51%) and SMPP (11.60%) decreased, while those of bronchiolitis (3.76%) and asthma (9.41%) increased. Compared with the 2017–2019 and 2020–2022 periods, in 2023, there was a significant increase in lobar pneumonia (21.50%) and SMPP (24.04%), and a decrease in asthma (2.11%) and bronchiolitis (1.79%).

#### Intrapulmonary and extrapulmonary complications in patients with SMPP

The number of patients with SMPP across the study period per year was 1,878 during 2017‒2019, 577 during 2020‒2022, and 2,869 in 2023 (Table 2). There were statistically significant differences between intra- and extrapulmonary complications in patients with SMPP. Intrapulmonary complications (plastic bronchitis, 421 [14.67%]; pulmonary artery embolism, 58 [2.02%]; and pleural effusion, 1,843 [64.24%]) and extrapulmonary complications (nervous system, 1,246 [43.43%] and circulatory system, 545 [19.00%]) significantly increased in patients with SMPP in 2023.

**TABLE 2:** Complications of patients with SMPP^*a*^

	2017–2019 (*n* = 1,878)	2020–2022 (*n* = 577)	2023 (*n* = 2,869)		
	*n* (%)	*n* (%)	*n* (%)	χ2	*P*
Intrapulmonary complications					
Plastic bronchitis	190 (10.12)_a_	45 (7.80)_a_	421 (14.67)_b_	47.788	<0.001
Pulmonary embolism	18 (0.96)_a_	8 (1.39)_a_	58 (2.02)_a_		
Necrotizing pneumonia	184 (9.80)_a_	78 (13.52)_b_	239 (8.33)_c_		
Pleural effusion	1,060 (56.44)_a_	299 (51.82)_a_	1,843 (64.24)_a_		
Extrapulmonary complications					
Nervous system	691 (33.79)_a_	194 (33.62)_b_	1,246 (43.43)_b_	44.335	<0.001
Circulatory system	309 (16.45)_a_	94 (16.29)_b_	545 (19.00)_a, b_		
Urinary system	6 (0.32)_a_	1 (0.17)_a_	10 (0.35)_a_		
Blood system	210 (11.18)_a_	98 (16.98)_b_	509 (17.74)_c_		
Skin and mucosal damage	719 (38.29)_a_	142 (24.61)_a_	1,068 (37.23)_a_		

^*a*^ Patients with SMPP were divided into three groups based on the study period: 1,878, 577, and 2,869 patients presented with SMPP annually during the 2017‒2019, 2020‒2022, and 2023 periods, respectively. Statistics on the number and proportion of intra- and extrapulmonary complications in patients with SMPP were obtained each year. A cross-tabulation statistical analysis was performed, and statistically significant differences were observed. The Bonferroni method was used for pairwise comparisons, and the differences between any two groups are shown. Each subscript (a, b, c) indicates a subset of the year category; the column proportions of these categories do not differ significantly from each other at the 0.05 level.

#### Bronchoscopic findings of children with SMPP

**Fig 6:**
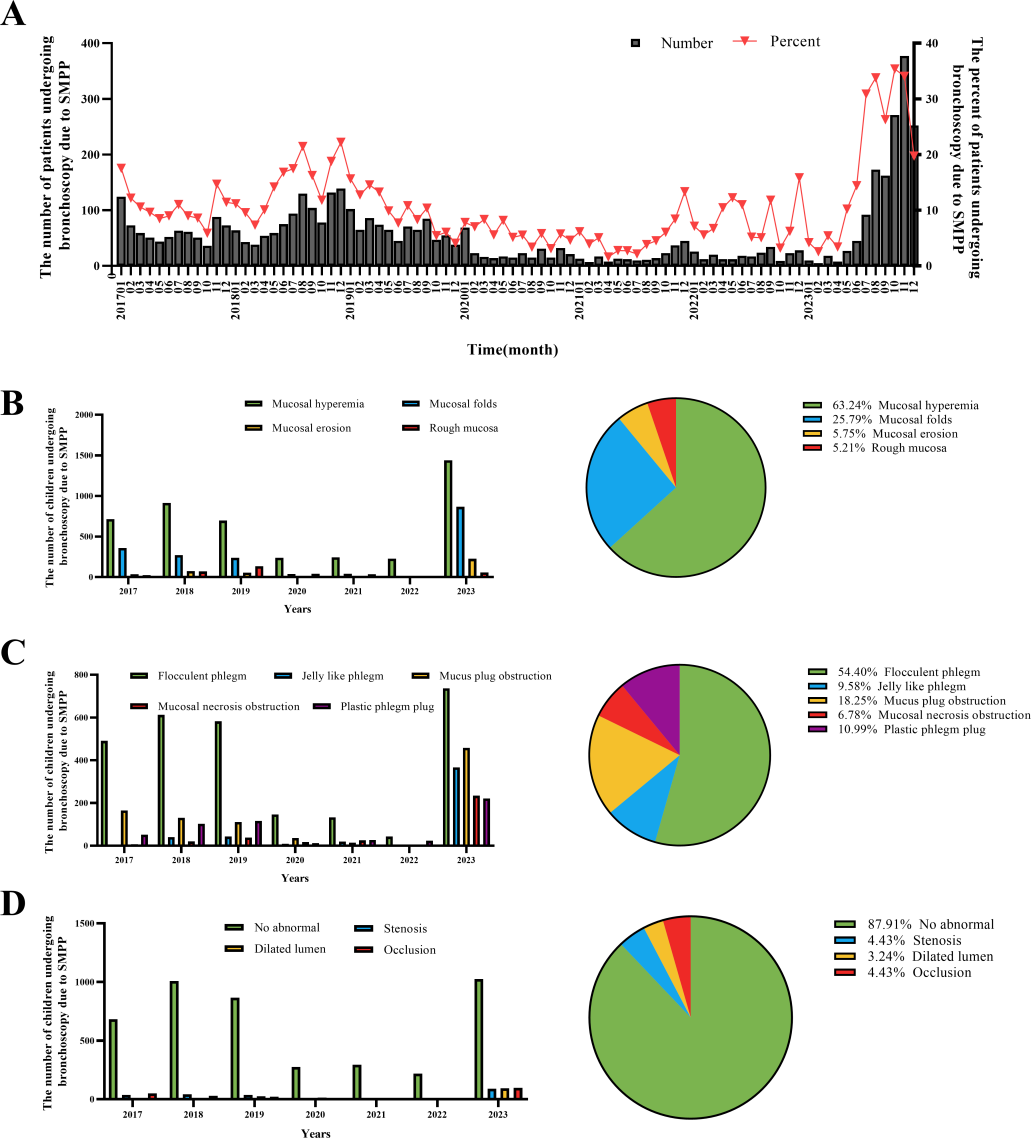
Patients who underwent bronchoscopy for SMPP. (**A**) Number and percentage of patients who underwent bronchoscopy for SMPP. (**B**) Bronchoscopic mucosal findings of MP pneumonia. (**C**) Bronchoscopic discharge manifestations of MP pneumonia. (**D**) Bronchoscopic manifestations of the bronchial wall in MP pneumonia.

The number of children with SMPP who underwent bronchoscopy was quantified to determine the incidence rate. The bronchoscopic manifestations were also statistically analyzed. A total of 37,183 patients in the NE region and 3,963 patients in the IM region were hospitalized with MP infection. Among them, 3,110 patients underwent bronchoscopy for SMPP in the NE group and 1,649 patients in the IM group; the mean percentage (number of patients who underwent bronchoscopy for SMPP/total number of patients who underwent bronchoscopy) was 11.57%. The number and percentage of patients who underwent bronchoscopy for SMPP were further analyzed by month (Fig. 6A). From January 2020, the number of patients who underwent bronchoscopy for SMPP declined sharply. In August and September 2023, the number and percentage of patients requiring bronchoscopy for SMPP increased significantly, indicating an epidemic trend. The number and proportion of patients with SMPP requiring bronchoscopy each year were 861 (11.52%) during 2017‒2019, 245 (5.85%) during 2020‒2022, and 1,440 (23.39%) in 2023 (Table 3).

**TABLE 3:** Bronchoscopic manifestations of patients with SMPP^*a*^

	2017–2019	2020–2022	2023		
	*n* (%)	*n* (%)	*n* (%)	χ2	*P*
Number and proportion of patients with SMPP who underwent bronchoscopy	861 (11.52)	245 (5.85)	1,440 (23.39)		
Mucosal manifestation					
Mucosal hyperemia	775 (64.85)_a_	236 (79.19)_b_	1,440 (55.56)_c_	183.985	<0.001
Mucosal folds	290 (24.27)_a_	29 (9.73)_b_	867 (33.45)_c_		
Mucosal erosion	54 (4.52)_a_	6 (2.01)_a_	226 (8.7)_b_		
Rough mucosa	76 (6.36)_a_	27 (9.06)_a_	59 (2.28)_b_		
Discharge manifestation					
Flocculent phlegm	562 (67.06)_a_	107 (62.57)_a_	736 (36.51)_b_	307.853	<0.001
Jelly-like phlegm	28 (3.34)_a_	11 (6.43)_a_	367 (18.20)_b_		
Mucus plug obstruction	136 (16.23)_a_	18 (10.53)_a_	458 (22.72)_b_		
Mucosal necrosis obstruction	22 (2.63)_a_	14 (8.19)_b_	234 (11.61)_b_		
Plastic phlegm plug	90 (10.74)_a_	21 (12.28)_a_	221 (10.96)_a_		
Lumen manifestation					
No abnormalities	852 (90.93)_a_	262 (92.25)_a_	1,025 (78.60)_b_	88.223	<0.001
Stenosis	39 (4.16)_a_	5 (1.76)_a_	89 (6.83)_b_		
Dilated lumen	12 (1.28)_a_	10 (3.52)_b_	93 (7.13)_b_		
Occlusion	34 (3.63)_a_	7 (2.46)_a_	97 (7.44)_b_		

^*a*^ Patients who underwent bronchoscopy were divided into three groups based on the study period: 861, 245, and 1,440 patients with SMPP underwent bronchoscopy every year during 2017‒2019, 2020‒2022, and 2023, respectively. Statistics on the number and proportion of bronchoscopic manifestations in patients with SMPP were obtained annually. A cross-tabulation statistical analysis was performed, and statistically significant differences were observed. The Bonferroni method was used for pairwise comparisons, and the differences between any two groups are shown. Each subscript (a, b, c) indicates a subset of the year category; the column proportions of these categories do not differ significantly from each other at the 0.05 level.

Most patients with SMPP had mucosal hyperemia (63.24%) and mucosal folds (25.79%) (Fig. 6B). The major bronchoscopic discharge manifestations in the SMPP cases were flocculent phlegm (54.40%), mucus plug obstruction (18.25%), and plastic phlegm plugs (10.99%) (Fig. 6C). Further analysis of bronchoscopic manifestations related to the bronchial wall in patients with SMPP revealed that most patients exhibited no abnormalities in the bronchial walls (87.91%). However, some patients exhibited bronchial stenosis (4.43%) or occlusion (4.43%) (Fig. 6D).

**Fig 7:**
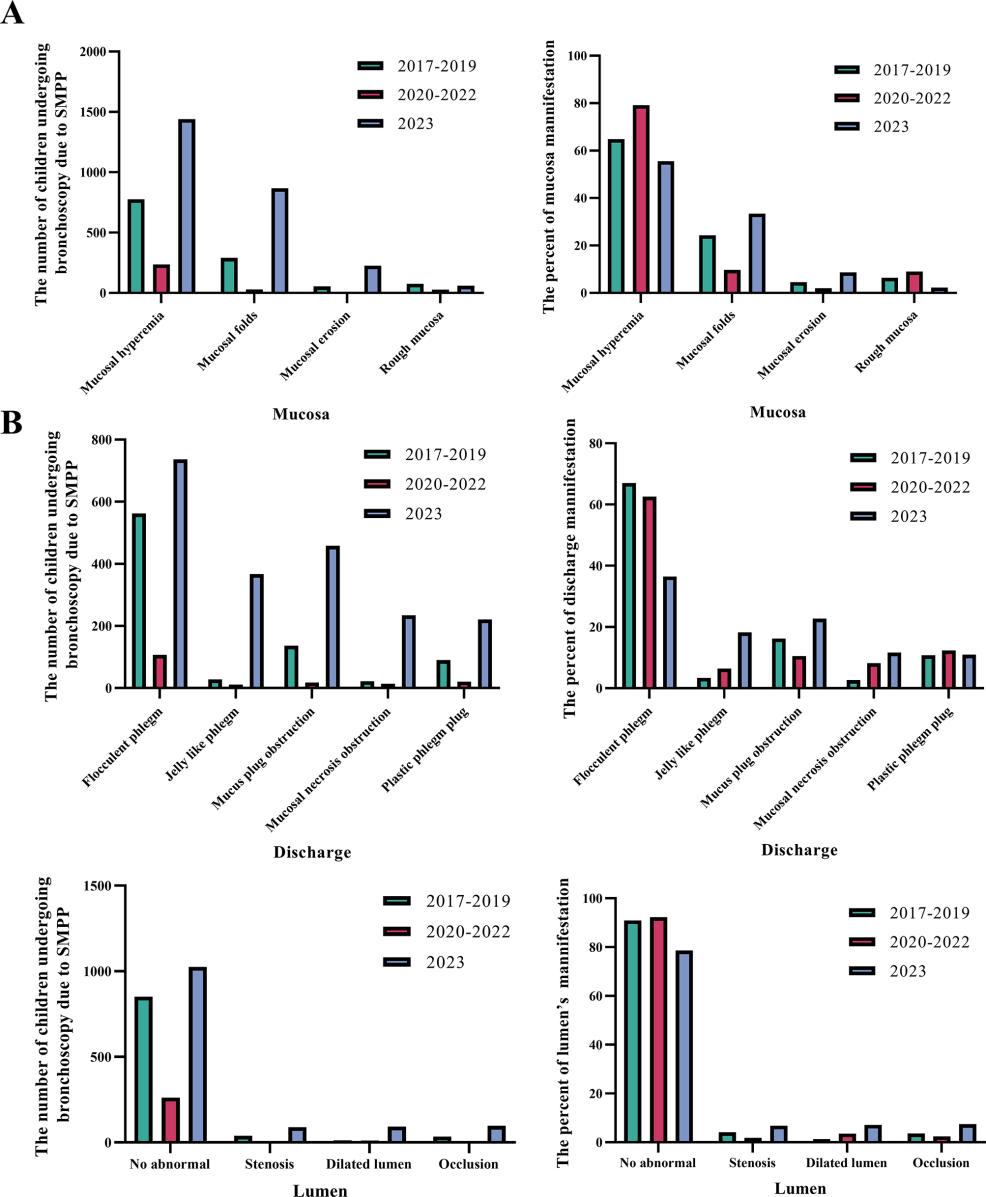
Patients who underwent bronchoscopy for SMPP were analyzed by different time periods. Number and percentage of (**A**) mucosa manifestations, (**B**) discharge manifestations, and (**C**) lumen manifestations in the 2017‒2019, 2020‒2022, and 2023 groups.

We then analyzed the changes in bronchoscopy performance each year (Fig. 7). Compared with that in the 2017‒2019 group, mucosal hyperemia under bronchoscopy in the 2020‒2022 group significantly increased (79.19%), while mucosal hyperemia (55.56%) and rough mucosa (2.28%) significantly reduced. Compared with that in the 2017‒2019 group, mucosal folds (33.45%) and mucosal erosion (8.7%) significantly increased in the 2023 group. Compared with that in the 2017‒2019 group, mucosal necrosis obstruction under bronchoscopy in the 2020‒2022 group significantly increased (8.19%), while flocculent phlegm (36.51%) significantly reduced. Compared with that in the 2017‒2019 group, jelly-like phlegm (18.20%), mucus plug obstruction (22.72%), and mucosal necrosis obstruction (11.61%) increased in the 2023 group; the differences were statistically significant. Analysis of luminal changes under bronchoscopy revealed that compared with that in the 2017‒2019 and 2020‒2022 groups, the number of abnormal luminal lesions, including stenosis (6.83%), dilated lumen (7.13%), and occlusion (7.44%), increased in 2023. These results suggest that the implementation of NPIs from 2020 to 2022 significantly reduced the number and proportion of bronchoscopies performed in patients with SMPP, with no significant changes in the severity of bronchoscopic manifestations. In the 2023 group, the number and proportion of bronchoscopies performed in patients with SMPP increased significantly; the severity of the bronchoscopic manifestations increased; and the proportion of jelly-like phlegm, mucus plug obstruction, and luminal problems increased.

## DISCUSSION

MP infections are prevalent in China (6, 7). However, previous studies on the epidemiological trend of MP infections in NE and IM are lacking. Therefore, in this study, we collected data on MP infection in these regions. In total, 5,593,530 children visited the outpatient and emergency departments, of which, 412,480 were hospitalized. The mean positivity rate for MP IgM in children who visited the outpatient and emergency departments and those who were hospitalized ranged from 7.80% to 10.12% and 27.18% to 30.10%, respectively.

In a previous study, MP infections were most commonly observed in the 5- to 9-year-old age group, while the prevalence of macrolide-resistant MP infections was higher among older children (8–10). In our study, the children hospitalized for MP infection were mainly in the 1- to 4- and 4- to 7-year-old age groups. Children aged 1 to 4 years are more likely to have aggravated clinical symptoms, including severe cough, wheezing, and dyspnea, which require hospitalization. Children aged 4 to 7 years had increased social activities and aggravated cross-infection. In most previous studies, the children were divided into those <5 years of age, those between 5 and 9 years of age, and those >17 years of age. The age span of the >5-year-old group was large; hence, the proportion of children with MP infection was larger than that of the young children group, which was the highest for children; the latter dropped as age increased, with an obvious descending turnpoint at 7 years old for MP infection (7).

Data from the Henan Children’s Hospital indicate that following the two waves of COVID-19 pandemic, there was a decrease in the number of positive results and in the positive rate of serological tests or MP RNA detection for several months (3). Subsequently, during the recovery period after the pandemic, a slight increase was observed, although these figures remained lower than those before the COVID-19 pandemic (3). MP and *Chlamydia pneumoniae* (CP) IgM antibodies were detected in all hospitalized children with acute respiratory tract infection at the Children’s Hospital Affiliated with Zhejiang University between January 2019 and December 2020. The implementation of several preventive and control measures against severe acute respiratory syndrome coronavirus 2 (SARS-CoV-2) during the COVID-19 pandemic not only helped contain the spread of SARS-CoV-2 but also sharply improved the incidence of other atypical pathogens, including MP and CP (11, 12). We studied the impact of NPIs on the epidemiology of MP before, during, and after their implementation. There was a decrease in the number of MP IgM-positive children in the outpatient and emergency departments, and a sharp decrease in the number of MP infections in hospitalized children from January 2020 to June 2023, consistent with previous studies (13–16). Considering the slow generation time (6 hours) and spread (1- to 3-week incubation period) of MP, a longer time interval may be required for their re-establishment within the population after discontinuing NPIs (5). Notably, the suppression of MP was sustained in 2021‒2022 after prolonged periods during which NPIs were relaxed or discontinued. Conversely, other pathogens such as influenza A and B, rhinovirus, and respiratory syncytial virus resurged, indicating community transmission (17, 18). Nevertheless, the percentage of hospitalized children with MP infections did not follow this trend, which differs from a study that suggested a decline in MP proportions during the post-COVID-19 period (19).

MP is a mucosal pathogen often present in the host respiratory tract (20). We collected data from hospitalized patients with MP infection and common respiratory diseases, including bronchopneumonia, lobar pneumonia, SMPP, asthma, bronchiolitis, and pertussis-like syndrome. In 2020, NPIs were implemented, which considerably decreased the number of patients with these diseases, consistent with the results of a previous study (21). Moreover, the number and proportion of children with SMPP who needed bronchoscopies increased sharply in October 2023, suggesting an outbreak of MP, with a significant increase in the proportion of severe infections. The diminished immune stimulation resulting from the decreased circulation of microbial agents and the associated decline in vaccine uptake created an “immunity debt,” potentially leading to adverse consequences upon the lifting of NPIs (22). Li et al. found that the delayed outbreak was related to the macrolide-resistant MP outbreak due to the A2063G mutation acquisition in the 23S rRNA (23).

MP mainly adheres to receptors on the membrane of airway epithelial cells through structures such as the P1 protein, thereby releasing toxic metabolites and causing epithelial cell damage (24). Persistent pathogenic bacteria result in prolonged pulmonary infection (25). Airway mucosal erosion, necrotic mucosal exfoliation, and sputum embolism are more common in refractory *Mycoplasma pneumoniae* pneumonia (RMPP) and SMPP than in common MPP (26, 27). These conditions may also lead to plastic bronchitis, lumen stenosis, and occlusion (28–30). Therefore, a bronchoscopy is important in the diagnosis and treatment of RMPP and SMPP (31). A previous study observed a correlation between RMPP and bronchiolitis obliterans (32), with some children exhibiting sputum casts during the second or third bronchoscopy (33). In our study, the number of patients who underwent bronchoscopy for SMPP declined sharply from January 2020 onward. The proportion of patients who underwent bronchoscopy for SMPP also declined considerably from June 2020 but increased sharply from September 2023; the complications and severity of SMPP also increased significantly. Most patients with SMPP exhibit mucosal hyperemia, mucosal folds, and flocculent phlegm. Some patients presented with mucus and plastic phlegm plugs. Furthermore, the bronchial walls of most patients showed no abnormalities; however, a small percentage exhibited stenosis and occlusion, warranting further analysis.

This was the first multicenter study on the prevalence of MP in the NE and IM regions of China. The number of patients was large, allowing us to study the epidemic trend of MP. However, this study has several limitations. First, the prevalence of macrolide resistance in the MP strains has not yet been determined. Second, only a few hospitals were included in this study, particularly in the Jilin and Heilongjiang regions. Third, we did not analyze the prognosis of SMPP with different bronchoscopic manifestations; only the bronchoscopic manifestations of SMPP were statistically analyzed.

Overall, MP infections are widespread in the NE and IM regions of China. After the implementation of NPIs, the number of children hospitalized with MP infections decreased; however, the proportion of hospitalized children infected with MP did not change significantly. Children hospitalized with MP infection were mainly concentrated in the 1- to 4- and 4- to 7-year-old age groups. Bronchopneumonia is the main manifestation of respiratory diseases caused by MP infection, accounting for 58.79% of cases. SMPP accounted for 18.43% of cases; the bronchoscopic findings were mostly mucosal hyperemia (63.24%), mucosal folds (25.79%), and flocculent phlegm (54.40%). Most children with SMPP showed no obvious damage to the bronchial wall; however, some children showed stenosis and occlusion during bronchoscopy. The positive rate of MP infection gradually increased since August and September 2023, with the number of severely ill children increasing significantly. When the NPIs were lifted, MP infections took on a state of a delayed outbreak epidemic, with the number and complications of SMPP surging.

## MATERIALS AND METHODS

### Study participants

This study comprised outpatients and inpatients aged 0–14 years old in the pediatric departments of six public Grade III hospitals between 1 January 2017 and 31 December 2023. The hospitals included Shengjing Hospital of China Medical University (National Center for Children’s Health, Northeast Region) (SJ), Dalian Women and Children’s Medical Center Group (DL), The First Affiliated Hospital of Jinzhou Medical University (JZ), The 2nd Affiliated Hospital of Harbin Medical University (HE), The Affiliated Hospital of Changchun University of Chinese Medicine (CC), and The Affiliated Hospital of Inner Mongolia Medical University (IMM). Data collection and analysis were conducted according to the regions where the hospitals were located, that is SJ, DL, JZ, CC, and HE in NE, and IMM in IM.

### Data collection

Hospitals from the NE (SJ, JZ, DL) and IM (IMM) regions provided data on pediatric outpatient and emergency visits, and hospitals from the NE (SJ, JZ, DL, HE, CC) and IM (IMM) regions provided data on hospitalizations from 1 January 2017 to 31 December 2023. The collected data included the total number of patients, number of patients with MP infection, patient age, disease spectrum, and bronchoscopic manifestations. The serum MP IgM test method is outlined in Table 4. The hospitalized patients were further divided into five age groups: 0- to 1-, 1- to 4-, 4- to 7-, 7- to 10-, and 10- to 14-year-old to analyze the age distribution of children with MP. All hospitals included in the study had laboratories that met the standards.

**TABLE 4:** Hospital conditions and serum MP IgM antibody test methods

Region	Hospital	Hospital level	Clinical laboratory	IgM test method (technique; product)	Detection instrumentation
Shenyang, Liaoning Province	Shengjing Hospital of China Medical University	National-level comprehensive Grade-A tertiary hospital	ISO15189	ELISA	Addcare ELISA1100, Shandong, China
Hohhot, Inner Mongolia Autonomous Region	The Affiliated Hospital of Inner Mongolia Medical University	National-level comprehensive Grade-A tertiary hospital	BSL-3	Magnetic particle chemiluminescence method	Automatic chemiluminescence immunoassay analyzer, Beijing, China
Changchun, Jilin Province	The Affiliated Hospital of Changchun University of Chinese Medicine	National-level comprehensive Grade-A tertiary hospital	ISO15189	ELISA	Radu RT-6100 Microplate Analyzer, Beijing, China
Dalian, Liaoning Province	Dalian Women and Children’s Medical Center Group	National-level comprehensive tertiary hospital	The Key Laboratory of Liaoning Province	Chemiluminescence method	Chemiluminescence tester iFlash 3000A, Guangdong, China
Jinzhou, Liaoning Province	The First Affiliated Hospital of Jinzhou Medical University	National-level comprehensive Grade-A tertiary hospital	The Key Laboratory of Liaoning Province	Chemiluminescence method	Chemiluminescence tester iFlash 3000A, Guangdong, China
Harbin, Heilongjiang Province	The 2nd Affiliated Hospital of Harbin Medical University	National-level comprehensive Grade-A tertiary hospital	The Key Laboratory of Heilongjiang Province	ELISA	Radu RT-6100 Microplate Analyzer, Beijing, China

Because China implemented NPIs from January 2020 to December 2022, we divided the data into three groups according to time: 2017‒2019 (before NPIs), 2020‒2022 (NPIs), and 2023 (after NPIs) to analyze and calculate the average number and proportion of children affected by MP each year.

SMPP often occurs in patients with MPP. The diagnostic criteria are based on the National Health Commission General Office’s Guidelines for the Diagnosis and Treatment of *Mycoplasma pneumoniae* Pneumonia in Children (2023 Edition) (34): (i) a sustained high fever (>39°C) for ≥5 days or fever for ≥7 days, with no downward trend in peak body temperature; (ii) wheezing, shortness of breath, dyspnea, chest pain, and hemoptysis, all of which are manifestations of severe diseases such as plastic bronchitis, asthma attacks, pleural effusion, and pulmonary embolism; (iii) extrapulmonary complications that do not reflect critical illness; (iv) a breathing air finger pulse oxygen saturation ≤0.93 in a resting state; (v) one of the following imaging manifestations: [a] a single lung lobe is involved in ≥2/3, with a uniform high-density consolidation, or two or more lobes have high-density consolidation (regardless of the size of the involved area), which may be accompanied by moderate to large amounts of pleural effusion or by symptoms of localized bronchiolitis; [b] diffuse bronchiolitis in one lung or ≥4/5 of both lung lobes may be combined with bronchitis and result in mucus plug formation and atelectasis; (vi) clinical symptoms progressively worsen and imaging shows that the lesions progress by more than 50% within 24–48 hours; and (vii) C-reactive protein, lactate dehydrogenase, and D-dimer levels are significantly elevated.

We statistically analyzed intrapulmonary complications (plastic bronchitis, pulmonary embolism, necrotizing pneumonia, and pleural effusion) and extrapulmonary complications (nervous system, circulatory system, urinary system, blood system, skin, and mucosal damage) in patients with SMPP, and the bronchoscopy findings of hospitalized children with SMPP. We analyzed the bronchoscopic findings and categorized them into three groups: mucosal manifestations (mucosal hyperemia, mucosal folds, mucosal erosion, and rough mucosa), discharge performance (flocculent phlegm, jelly-like phlegm, mucus plug obstruction, mucosal necrosis obstruction, and plastic phlegm plug), and lumen changes (no abnormalities, stenosis, dilated lumen, and occlusion).

### Statistical analysis

All statistical analyses were performed using SPSS version 27.0. Pearson’s χ^2^ was employed to compare the categorical variables between the groups, with statistical significance defined as *P*  <  0.05.

## Data Availability

The data generated in this study are available from the corresponding author upon reasonable request.
